# Explaining the social gradient in sickness absence: a study of a general working population in Sweden

**DOI:** 10.1186/1471-2458-13-545

**Published:** 2013-06-05

**Authors:** Jesper Löve, Gunnel Hensing, Kristina Holmgren, Kjell Torén

**Affiliations:** 1 Department of Public Health and Community Medicine, The Sahlgrenska Academy, University of Gothenburg, Göteborg, Sweden

## Abstract

**Background:**

Some previous studies have proposed potential explanatory factors for the social gradient in sickness absence. Yet, this research area is still in its infancy and in order to comprise the full range of socioeconomic positions there is a need for studies conducted on random population samples. The main aim of the present study was to investigate if somatic and mental symptoms, mental wellbeing, job strain, and physical work environment could explain the association between low socioeconomic position and belonging to a sample of new cases of sick-listed employees.

**Methods:**

This study was conducted on one random working population sample (n = 2763) and one sample of newly sick-listed cases of employees (n = 3044), drawn from the same random general population in western Sweden. Explanatory factors were self-rated 'Somatic and mental symptoms', 'Mental well-being', 'job strain', and 'physical work conditions' (i.e. heavy lifting and awkward work postures). Multiple logistic regression analyses were used.

**Results:**

Somatic and mental symptoms, mental well-being, and job strain, could not explain the association between socioeconomic position and sickness absence in both women and men. However, physical work conditions explained the total association in women and much of this association in men. In men the gradient between Non-skilled manual OR 1.76 (1.24;2.48) and Skilled manual OR 1.59 (1.10;2.20), both in relation to Higher non-manual, remained unexplained.

**Conclusions:**

The present study strengthens the scientific evidence that social differences in physical work conditions seem to comprise a key element of the social gradient in sickness absence, particularly in women. Future studies should try to identify further predictors for this gradient in men.

## Background

There is abundant scientific evidence for an association between lower socioeconomic position and higher rates of sickness absence [1-9]. Previously, physical and psychosocial work conditions, health-related behaviors, and different health outcomes have been suggested as explanatory factors. The explanatory effect of health-related behavior [1,3], psychosocial work factors [1,2,7] and self-rated general health seem to be small or even inconclusive [2,7,10]. Recently two studies in unison concluded that physical work conditions were the strongest explanatory factor for occupational class differences in sickness absence. However, in neither of these studies was the association totally explained by this factor [1,3], and only one of them used a random population sample [1]. In a recent study, using the same random working population sample as the present study, physical work ability explained the total association between socioeconomic position and sickness absence in women. Yet, some of this association remained unexplained in men [11].

It is important to note that the social gradient in sickness absence may vary by duration and pattern of sickness absence [7], tends to be stronger in men [1,2,7], and may vary by clinical diagnosis [12]. To include the full range of socioeconomic positions there is also a need for more studies on random working populations in order to explain the social gradient of sickness absence [2].

The main aim of the present study was to investigate if the social gradient in sickness absence in women and men could be explained by age, somatic and mental symptoms, mental well-being, psychosocial work conditions (i.e. job strain), and physical work conditions (i.e. heavy lifting and awkward working postures). A second aim was to examine if the explanatory power of these factors differed between women and men.

## Methods

This study comprised one random population sample and one sample of new cases of sick-listed employees, 19–64 years old. Both samples were drawn from the population of the Västra Götaland region of western Sweden, inhabiting approximately 1.6 million people and including both urban and rural areas. The random population sample was randomly selected by Statistics Sweden. The Swedish Social Insurance Agency identified the sick-listed sample as all employer-reported new cases of sick-leave (i.e. >14 days), during a period from the 18^th^ of February 2008 to the 15^th^ of April 2008. Consequently, these individuals became sick-listed in a new sick-leave spell and none of them had any earlier sick-leave spell the year under study. Baseline data was then collected from the 15^th^ of April 2008 to the 30^th^ of June 2008. The response rates were 57% in women (n = 2,234) and 44% in men (n = 1,793) in the random working population sample, and 58% in women (n = 2,196) and 47% in men (n = 1,114) in the sick-listed sample. More specific details on the sampling procedure and the characteristics of these samples can be found elsewhere [11]. The Health Assets Project was approved by the Ethics Committee, University of Gothenburg, Sweden.

### Explanatory variables

Six categories of occupational position [13] were used to assess socioeconomic position: higher non-manual employees, intermediate non-manual employees, lower non-manual employees, skilled worker, unskilled worker, entrepreneurs. Because only three individuals were classified as being entrepreneurs these individuals were included in the higher non-manual employees’ category.

*Age* was measured as a continuous variable provided by Statistics Sweden. Since it is possible that the association between SEP and sickness absence may follow different patterns due to age groups this variable was inserted as a potential confounder in all five models.

*Physical and mental symptoms* were measured with a symptom checklist. The included symptoms were all used as indicators of perceived health and covered a number of symptoms that are commonly reported by both women and men in the general population [14]. This procedure is in line with a previous study supporting an interpretation of these common symptoms as a single entity phenomenon mirroring general distress [15]. The checklist covered stomach pain, palpitations, breathing disorders, fatigue, dizziness, headache, chest pain, low back pain, shoulder and neck pain, difficulties falling asleep, frequent awakenings, and difficulties concentrating. Internal consistency (Cronbach’s alpha) of these 12 symptoms was 0.82 in the present sample. The symptoms were all measured with the following question, “How often have you had the following symptoms during the past 12 months?” with the response alternatives “Almost never or never”, “Now and again during the months”, “Now and again during the week”, and “Nearly every day”. The response alternatives were graded between 0 and 3 points. Consequently, the scale ranged from 0–36 points, which were divided into quartiles in the total cohort; very low level of symptoms (0–3 points), low level (4–6 points), high level (7–11 points), and very high level of symptoms (12–36 points). The validity of this measurement was previously shown to be satisfactory [15].

*Mental well-being* was measured with the WHO-10 Well-being Index [16]. It comprised the question, “How have you felt during the past week?” followed by ten statements like, “I have felt sad and down”, “… energetic, active, and optimistic go-ahead”, and “Life is full of interesting things”, which the respondent had to grade on an ordinal scale from “All the time” to “Never”. The answers were then graded from 0 to 3 points resulting in a scale from 0 to 30 points. The Scale was then divided into quartiles: “Very good mental health” (22–30 points), “Good mental health” (18–21 points), “low mental health (13–17 points), and “Very low mental health” (0–12 points). In order to ease the interpretation of the results the variable was reversed and thus calculated and presented as *low mental well-being.*

*Job demands and job control (i.e. job strain)* was measured by five and six questions [17], respectively, with four response alternatives ranging from “No, never” to “Yes, frequently”. The answers were graded from one giving a sum of 5–20 points for job demands, and 6–24 points for job control. Job strain was defined as having unfavorable (high) job demands (i.e. over median, 14) in combination with unfavorable (low) control (i.e. under median, 18). All other combinations of demands and control, including levels equal to median values were defined as non-job strain [18].

*Physical work environment* was measured with two items, “Does your work require heavy lifting?” and “Do you work in a crooked, twisted or otherwise unsuitable working posture?” with four response alternatives ranging from “Yes, frequently” to “No, never/almost never”.

### Outcome variables

The main outcome was defined as the odds ratio of belonging to the sample of newly sick-listed cases with the random working population sample as reference.

### Statistical analyses

A series of logistic regression analyses were calculated in the total population and in women and men, respectively, in order to examine the explanatory effect of included factors. Odds ratios (OR) and 95% Confidence intervals were calculated using the LOGISTIC procedure in SAS. Socioeconomic position was inserted as a CLASS variable resulting in a point estimate for all socioeconomic position levels respectively, with higher non-manual as reference. First, the association between socioeconomic position and belonging to the sick-listed sample was adjusted by age. Thereafter, somatic and mental symptoms, mental well-being, job strain, and physical work conditions, were added in four separate models together with age. The explanatory effect of these factors was then evaluated through comparing the size of the point estimates (OR) and width of the confidence interval with the point estimates of the unadjusted analyses. A confidence interval covering 1 was interpreted as a statistically insignificant association. Potential multicollinearity between the covariates was tested through analyses of correlation and Variance Inflation Factor (VIF). According to Kleinbaum et al. (1998) a role of thumb is that a VIF >10 may be problematic when it comes to a multiple regression [19].

All calculations were run using SAS version 9.2 (SAS Institute, Cary, NC).

## Results

In comparison with the random working population sample the sick-listed sample had a higher proportion of older people, people in lower socioeconomic positions, and people that were civil servants. In the sick-listed sample, there was also a higher proportion of people with physical and mental symptoms, low mental well-being, high job strain, and problematic physical work environment. This was true in both women and men except for employer where no difference between the samples was observed among men (see Table 1).

**Table 1:** Characteristics of the participants in the random working population sample and the sick-listed sample (n = 5807), for women and men, respectively

	**Women**			**Men**		
	**n = 3547**			**n = 2260**		
	**Sick-listed sample**	**Pearson’s Chi square**^**1**^	**Random working population sample**	**Sick-listed sample**	**Pearson’s Chi square**^**1**^	**Random working population sample**
	**n = 2022**		**n = 1525**	**n = 1022**		**n = 1238**
		**Chisq.**			**Chisq.**	
	**% (n)**	**Value**	**% (n)**	**% (n)**	**Value**	**% (n)**
**Years old**						
19 – 30	10 (208)	23.91*	15 (224)	12 (125)	47.75*	17 (215)
31 – 50	47 (953)		49 (751)	40 (412)		49 (610)
51 – 64	43 (861)		36 (550)	48 (485)		34 (413)
**Land of birth**						
Nordic countries	92 (1857)	3.23	93 (1425)	88 (896)	7.10*	91 (1128)
Other	8 (165)		7 (100)	12 (126)		9 (110)
Socioeconomic position						
Higher non-manual	11 (227)	86.89*	16 (234)	11 (106)	101.23*	21(257)
Intermediate non-manual	27 (550)		30 (447)	16 (158)		24 (290)
Lower non-manual	15 (291)		18 (265)	7 (67)		9 (290)
Skilled manual	21 (425)		17 (252)	29 (288)		21 (249)
Non-skilled manual	26 (511)		20 (303)	38 (383)		26 (312)
**Employer**						
Private/self-employed	30 (567)	36.52*	40 (576)	71 (672)	3.20	73 (856)
Public	70 (1332)		60 (855)	29 (269)		27 (317)
**Physical and mental symptoms**						
Very low level	11 (202)	171.75*	21 (288)	23 (215)	109.94*	34 (365)
Low level	19 (348)		27 (373)	22 (207)		30 (326)
High level	29 (552)		30 (415)	26 (243)		24 (267)
Very high level	41 (763)		22 (294)	29 (272)		12 (127)
**Mental well-being**						
Very good	18 (325)	82.28*	25 (363)	23 (212)	77.57*	30 (345)
Good	25 (461)		32 (458)	24 (218)		35 (407)
Low	26 (477)		24 (341)	27 (247)		22 (258)
Very low	31 (564)		19 (269)	26 (241)		13 (158)
**Job strain**						
Low	80 (1613)	47.63*	88 (1349)	83 (844)	45.27*	92 (1138)
High	20 (409)		12 (176)	17 (178)		8 (100)
**Heavy lifting**						
No, never/almost never	23 (459)	75.36*	32 (483)	16 (164)	107.51*	31 (383)
No, seldom	19 (387)		24 (354)	18 (178)		23 (276)
Yes, sometimes	25 (491)		23 (350)	29 (288)		25 (312)
Yes, frequently	33 (656)		21 (320)	37 (367)		21 (255)
**Awkward working postures**						
No, never/almost never	20 (397)	90.79*	27 (412)	16 (153)	115.49*	31 (374)
No, seldom	19 (384)		25 (376)	19 (191)		24 (300)
Yes, sometimes	27 (531)		27 (413)	28 (279)		24 (296)
Yes, frequently	34 (673)		20 (303)	37 (371)		21 (256)

*Statistically significant difference (p < .05), between the sick-listed sample and the random working population sample.

^1^ Chi square value for the distributional comparison between the sick-listed sample and the random working population sample.

All potential explanatory factors showed positive bivariate associations with belonging to the sample of newly sick-listed cases. No problems with multicollinearity between the variables were noted. There was an evident association between lower socioeconomic position and belonging to the sample of newly sick-listed cases in both women and men when adjusting for age (i.e. Model 1). In men the Odds ratios increased for each downwards step in socioeconomic position, OR 1.35 (1.00:1.78), OR 1.70 (1.16–2.51), OR 3.25 (2.43–4.35), and OR 3.34 (2.53–4.40). This stepwise gradient was less prominent in women. Adjusting for somatic and mental symptoms (i.e. Model 2) resulted in very small changes in both point estimates and confidence intervals in both women and men. Yet, in men the association between lower non-manual and higher non-manual became statistically insignificant OR 1.51 (95% CI 0.99:2.30). Even the explanatory effect of the mental well-being index (i.e. Model 3) and psychosocial work conditions (i.e. Model 4) resulted in hardly any or small changes in point estimates or confidence intervals. Nevertheless, when entering physical work conditions (i.e. Model 5) for women, all point estimates were very close to one and all associations became statistically insignificant. Even in men, physical work conditions had a strong explanatory effect on the differences between the different socioeconomic position-groups. Yet, although the point estimates were clearly reduced in all associations in Model 5 the gradient between Non-skilled manual OR 1.76 (1.24:2.48), and Skilled manual OR 1.59 (1.10:2.30), in relation to Higher non-manual, remained unexplained by the included variables (see Table 2). Since it was noted that a potential bias due to multicollinearity could be at hand due to high correlation (.75) between the two items covering physical work demands the model was also ran with these two measures separately. However, no bias was observed.

**Table 2:** Logistic mutiple regressions between lower levels of socioeconomic position and belonging to the sick-listed sample, with higher non-manual as reference

	**Women**					**Men**				
	**(n = 3547)**					**(n = 2260)**				
	***Model 1***	***Model 2***	***Model 3***	***Model 4***	***Model 5***	***Model 1***	***Model 2***	***Model 3***	***Model 4***	***Model 5***
	**Adjusted for age**	**Adjusted for age, somatic and mental symptoms**	**Adjusted for age and mental well-being (WHO)**	**Adjusted for age and job strain**	**Adjusted for age and physical work conditions**	**Adjusted for age**	**Adjusted for age, somatic and mental symptoms**	**Adjusted for age and mental well-being (WHO)**	**Adjusted for age and job strain**	**Adjusted for age and physical work conditions**
	**OR (95% CI)**	**OR (95% CI)**	**OR (95% CI)**	**OR (95% CI)**	**OR (95% CI)**	**OR (95% CI)**	**OR (95% CI)**	**OR (95% CI)**	**OR (95% CI)**	**OR (95% CI)**
Higher non-manual	1.00	1.00	1.00	1.00	1.00	1.00	1.00	1.00	1.00	1.00
Intermediate non-manual	1.27 (1.01:1.58)	1.38 (1.09:1.75)	1.31 (1.04:1.66)	1.24 (0.99:1.55)	1.00 (0.79:1.26)	1.35 (1.00:1.83)	1.21(0.88:1.68)	1.25 (0.91:1.71)	1.33 (0.98:1.80)	1.15 (0.85:1.57)
Lower non-manual	1.12 (0.87:1.43)	1.09 (0.83:1.42)	1.16 (0.90:1.51)	1.03 (0.81:1.33)	1.00 (0.78:1.29)	1.70 (1.16:2.51)	1.51 (0.99:2.30)	1.70 (1.14:2.54)	1.64 (1.11:2.43	1.27 (0.85:1.91)
Skilled manual	1.74 (1.36:2.10)	1.61 (1.25:2.09)	1.77 (1.38:2.28)	1.61 (1.26:2.05)	0.99 (0.74:1.33)	3.25 (2.43:4.35)	3.09 (2.26:4.22)	3.20 (2.36:4.33)	3.12 (2.33:4.18)	1.59 (1.10:2.30)
Non-skilled manual	1.79 (1.42:2.26)	1.70 (1.32:2.18)	1.82 (1.42:2.33)	1.58 (1.25:2.00)	1.05 (0.79:1.39)	3.34 (2.53:4.40)	2.88 (2.14:3.88)	3.01 (2.25:4.01)	3.00 (2.27:3.97)	1.76 (1.24:2.48)
Age	1.02 (1.01:1.02)	1.02 (1.01:1.02)	1.02 (1.01:1.02)	1.02 (1.01:1.02)	1.02 (1.01:1.03)	1.03 (1.03:1.04)	1.03 (1.02:1.04)	1.04 (1.03:1.04)	1.04 (1.03:1.04)	1.04 (1.03:1.04)
Phys. & mental symptoms		1.57 (1.46:1.68)					1.41 (1.29:1.54)			
Low mental well-being			1.35 (1.26:1.44)					1.26 (1.06:1.50)		
Job strain				1.88 (1.54:2.29)					2.21 (1.68:2.92)	
Heavy lifting					1.15 (1.04:1.27)					1.16 (1.00:1.32)
Awkward working postures					1.20 (1.09:1.31)					1.28 (1.12:1.47)

Odds ratios (OR) with 95% Confidence intervals (CI) adjusted for age are presented in *Model 1*. The explanatory effect of physical and mental symptoms is examined in *Model 2,* of mental well-being in *Model 3,* of mental work environment (i.e. job strain) in *Model 4,* and of physical work environment in *Model 5.*

The distributional relationship between socioeconomic position and the explanatory key variables showed that there were fewer younger workers (i.e. 19–30 years) in higher non-manual positions than in non-skilled manual positions. The proportion reporting heavy lifting was very low in higher non manual positions and very high in skilled and non-skilled manual positions. However, in intermediate non-manual positions the proportion reporting heavy lifting were 33% in women and 19% in men. A similar pattern was apparent in awkward working postures with 42% women and 20% men in intermediate non-manual position. In men the proportion reporting physical and mental symptoms was somewhat higher in lower socioeconomic positions. Yet, in women this difference between socioeconomic positions was almost non-existent (see Table 3). To investigate the unique explanatory effect of physical work conditions in relation to self-reported physical work ability (details can be found elsewhere [11]) some additional analyses were conducted in men. When physical work ability was entered into a model alone (i.e. together with age) Odds ratios were OR 2.32 (1.73:3.11) for Non-skilled manual and OR 2.42 (1.78:3.28) for Skilled manual in relation to Higher non-manual. This was then compared with the associations in Model 5 above where work conditions alone attenuated the Odds ratios to OR 1.76 (1.24:2.48) for Non-skilled manuals and OR 1.59 (1.10:2.30) for Skilled manuals. The lower explanatory power of physical work ability in comparison with physical work conditions was also observed when entering physical work ability into Model 5 (see above) resulting only in a slight attenuation of the associations with OR 1.53 (1.08:2.19) for Non-skilled manuals, and OR 1.50 (1.02:2.19) in Skilled manual in relation to Higher non-manual. Hence, the unique explorative effect was clearly higher from work conditions than from physical work ability. Entering land of birth and employer into the same model did not attenuate the point estimates any further.

**Table 3:** Distribution of socioeconomy in relation to age group, heavy lifting, working posture, and mental and physical symptoms

	**Women (n = 1525)**								
**Socioeconomic position**	**Years old , %(n)**			**Heavy lifting, %(n)**		**Awkward working postures, %(n)**		**Physical and mental symptoms, %(n)**	
	**19-30**	**31-50**	**51-64**	**Yes**	**No**	**Yes**	**No**	**High**	**Low**
Higher non-manual	9 (20)	60 (141)	31 (73)	2 (5)	98 (229)	14 (32)		51 (108)	49 (105)
Intermediate non-manual	12 (52)	52 (232)	36 (163)	33 (146)	67 (298)	42 (187)	58 (255)	45 (176)	55 (213)
Lower non-manual	14 (37)	49 (129)	37 (99)	18 (48)	82 (213)	23 (60)	77 (201)	55 (133)	45 (107)
Skilled manual	15 (37)	45 (114)	40 (101)	87 (216)	13 (32)	80 (198)	20 (50)	55 (128)	45 (104)
Non-skilled manual	25 (77)	41 (123)	34 (103)	82 (242)	18 (54)	78 (230)	22 (65)	57 (158)	43 (120)
	**Men (n = 1238)**								
	**19-30**	**31-50**	**51-64**	**Yes**	**No**	**Yes**	**No**	**High**	**Low**
Higher non-manual	9 (23)	54 (139)	37 (95)	6 (15)	94 (242)	8 (20)	92 (237)	29 (63)	71 (155)
Intermediate non-manual	10 (28)	54 (157)	36 (105)	19 (55)	81 (233)	20 (57)	80 (231)	36 (93)	64 (169)
Lower non-manual	27 (28)	43 (46)	30 (32)	33 (35)	67 (70)	44 (46)	56 (59)	36 (33)	64 (58)
Skilled manual	24 (60)	46 (114)	30 (75)	87 (212)	13 (33)	82 (202)	18 (43)	36 (77)	64 (135)
Non-skilled manual	23 (72)	46 (144)	31 (96)	77 (237)	23 (70)	70 (215)	30 (92)	41 (115)	59 (165)

For the random working population sample for women and men respectively.

## Discussion

Surprisingly, in the present study the explanatory effect of somatic and mental symptoms, mental well-being, and job strain, on this social gradient of sickness absence, was small and in some cases even non-existent. This was true for both women and men. However, physical work conditions (i.e. heavy lifting and awkward working postures) explained the entire association between socioeconomic position and sickness absence in women. Not only did the associations become statistically insignificant but also the point estimates were all close to one. Although physical work conditions had a strong explanatory effect even in men the association between the two manual levels and the Higher non-manual level in men remained unexplained. While the previous study on the present material emphasized the explanatory effect of self-reported physical work ability for the social gradient in sickness absence [11] it left us with the query of whether it was the individual resources (e.g. health) or the work environment that constituted the critical element in this effect. In the additional analyses of the present study, in men, we observed that although self-reported physical work ability had an explanatory effect alone, the unique effect of physical work conditions was clearly more important. Since physical work ability [11] and physical work conditions respectively explained the total associations in women, in the present sample, similar analyses was not conducted in this group. Hence, together with previous studies [1-3] the present study strengthens scientific evidence suggesting that individual resources and psychosocial work conditions may not have a large impact on the social gradient in focus, but are still important factors in sickness absence per se. That physical work conditions (i.e. ergonomic) had the strongest explanatory effect on the social gradient in sickness absence is in line with three recent studies [1-3] of which one was performed in a general working population in Denmark [1]. Hereby, the present results support the idea that physical work conditions are the main explanatory factor for the social gradient in sickness absence. It is of particular importance that this result is observed in a random working population. The reason for this is that such a sample provides the full range of socioeconomic positions, which are not found in more specific workplace samples. One of the strengths of this study is also that the measure of sickness absence was not based on self-reports but on new cases of sick-listed employees (>14 days), reported by the employer.

The present methodology may bring about a concern of circulatory reasoning. That is, that socioeconomy and physical work conditions end up representing the same phenomena. However, this should have been a graver problem if the aim had been to explain the distribution in one cohort with a variable strongly correlated with how this distribution was accomplished in the first place. As we, in the present study, compared the distribution of socioeconomy in two different cohorts, finding that this difference was explained by physical work conditions, the issue of circulatory reasoning is not applicable in the same matter. Furthermore, although the proportional distribution (Table 3 above) showed an association between socioeconomy and physical work conditions neither the correlation matrix nor the Variance Inflation Factors indicated any worrying covariance (i.e. multicollinearity) between these two variables. Although one could expect a high correlation between socioeconomic position and physical work conditions many occupations labeled as manual are not very physically demanding as captured in the two items used in the present study, e.g. observational occupations in the industry. There are also non-manual occupations that actually comprise heavy lifting and particularly awkward working postures, e.g. nurses. Hence, the relation between socioeconomic position and physical work conditions is today more complex than previously.

Somatic and mental health symptoms had low explanatory effect in both women and men which might appear as somewhat odd since an individual’s health position is conceptually seen as one of the main constituents for being sick-listed. One could also assume that the previously well-supported social gradient in health [20] would play a major role even in the social gradient in sickness absence. Still, the present study, in line with some previous, observed only small or even inconclusive results for the explanatory effect of self-rated general health on the social gradient of sickness absence [2,7,10]. However, a health problem per se is not a sufficient reason for sick-leave in the Swedish and most other welfare state regulations. In Sweden, in order to fulfill the criteria for sick leave (i.e. >14 days) one’s work ability has to be reduced due to a clinical diagnosis. This may explain why physical and mental health symptoms did not attenuate the social gradient in sickness absence in this study. Yet, in order to state that socioeconomic health differences do not contribute to the social gradient in sickness absence future studies should involve more objective measurements of health. One example could be to investigate whether socioeconomic differences in the prevalence of specific clinical diagnoses could work as an explanation in this issue, particularly since the association between socioeconomy and sickness absence might be diagnose-specific [12].

Unfortunately, we did not have access to any information about occupation. Still, we must recognize that the labor market in Sweden is highly segregated between women and men where about 90% of all health care personnel are women [21]. Consequently, it would not be surprising if the explanatory factors for the social gradient in sickness absence would differ between women and men. For example, one reason for physical work conditions having such a strong explanatory effect on the social gradient specifically in women may be the so-called horizontal segregation. Many women in lower socioeconomic groups work within the health care sector where ergonomic exposures like lifting and awkward working postures are common [22]. It is also plausible that the historical development of ergonomic assistance has been more successfully implemented in male-dominated occupational groups like industrial or construction workers compared to care organizations that generally include more women. A previous study observed that assistant nurses have a six fold higher risk of over-exertion back injuries compared to other employed women in Sweden [23]. Consequently, it is possible that by solely focusing the measurement of physical exposure on heavy lifting and awkward working postures that may be essential predictors of the social gradient in sickness absence among women, exposures more prevalent in occupations dominated by men were left out. Eng and colleagues (2011) recently observed that male workers are two to four times more likely to report exposure to dust, chemicals, load noise, irregular hours, and vibrating tools than female workers. Even when comparing women and men with the same occupations clear gender differences in exposures are observed [24].

Furthermore one must recognize that in the present study, like previous studies (e.g. [2]), the differences between socioeconomic groups regarding sickness absence were less pronounced among women. One reason for the smaller gradient in women may be that work-related mental disorders due to stress, where the social gradient is reversed, are more common in women. This reversed social gradient is also steeper in women [25]. Correspondingly, Lahelma and colleagues (2005) observed a social gradient in global and physical health but not in mental health [26]. Another reason for the less pronounced social gradient in women could be that measures of socioeconomic position may have less precision in women since they fail to capture significant elements of gendered structures including the distribution not only over different types of occupations discussed above but also the distribution of management positions and responsibilities of the unpaid work [27-29].

One surprising finding was that although having socioeconomic position in the same models ‘physical and mental symptoms’, ‘low mental well-being’, ‘jobs strain’, and ‘physical work exposures’ were associated with sickness absence, in both women and men. This observation further implies the complexity between the social gradient of sickness absence and its potential explanations.

Acknowledging the recognized difficulties in persistently changing individual health-related behavior and psychosocial work conditions, the present result does in fact leave us with a relatively attainable goal of reducing the socioeconomic differences in physical work conditions. Yet, in order to provide better knowledge for interventional design future studies should be more specific and discriminate between the importance of different dimensions of the physical work environment in relation to socioeconomy and sickness absence.

It is important to recognize that the overall response rate in the present study was rather low (i.e. 52%). The analysis on non-respondents (figures not shown) showed that the proportions of men, younger individuals, individuals with the lowest income level, and individuals born outside the Nordic countries were lower than in the total population. Although the overrepresentation of these groups in the dropouts is a limitation that this study shares with most population-based studies it must be assumed that these groups may be highly important in studies investigating health in relation to socioeconomic circumstances. However, the specific patterns according to age, income and gender of the non-respondents were quite similar across the both samples that make comparisons possible. That we have a lower response rate in individuals with the lowest income level and individuals born outside of the Nordic countries could have resulted in somewhat lower Odds ratios. Yet, the relational patterns would probably be very similar. The drop-out from younger individuals may not have had a major impact on the results since long-term sickness absence is rare in this group. However, since the data collection was based on postal questionnaires no information on potential differences in health was available. Finally, although constituting a type of case–control design, the data used in the present study are collected at one point in time with the limitations that this design brings.

Not being a traditional longitudinal study it could be stated that sick-listed individuals with for example lower back pain may report differently on items capturing physical work environment. However, since back pain is rather common even in the general population potential reporting bias should be quite similar in the two samples although individuals in the sick-listed sample may have had more acute problems. The present analyses were also adjusted for both physical and mental symptoms.

The results of the present study emphasize that even in a so-called post-industrial and high-income country like Sweden physical work conditions like heavy lifting and awkward working postures still seem to play a very special role in the relation between socioeconomy and sickness absence. Beyond the use of more sophisticated data materials and analyses (e.g. [30]) future studies should dig deeper into what predictors may affect this association in men but also into the importance of socioeconomic differences regarding the prevalence of specific diagnoses. Finally, it should also be investigated whether the predictors of sickness absence may act in divergent patterns in the different socioeconomic groups.

## Conclusions

In the present study, physical work exposure (i.e. ergonomic) had the highest explanatory power for the social gradient in sickness absence in both women and men. However, in women physical work exposures explained the total association whereas the gradient between manual and non-manual socioeconomic groups remained unexplained in men. Self-reported somatic and mental symptoms, mental well-being, and job strain had very little or no explanatory power. Observing these results in a random working population sample, using newly sick-listed cases, is of particular importance since such a sample provides the full range of socioeconomic positions not found in more specific workplace samples.

Although a bulk of literature emphasize the importance of psychosocial work conditions in relation to sickness absence the present study strengthen the scientific evidence that in relation to the social gradient in sickness absence physical work conditions seem to comprise the key element. That the significance of physical work environment is observed in high-income countries, sometimes called post-industrial societies, remind us of the different living conditions that are still prevailing in these societies. Still, this relation needs further attention, particularly in examining what conditions may affect the social gradient in sickness absence among men, and whether the predictors of sickness absence may diverge between different socioeconomic groups.

## Abbreviations

OR: Odds ratios; CI: Confidence intervals.

## Competing interests

The authors declared that they have no competing interest.

## Authors’ contribution

GH is the main investigator of the Health Assets Project responsible for the idea, the overall design and content of the questionnaire. KH has been project leader active in all parts of the including questionnaire construction and data collection and management. In this particular study all authors were involved in the study design. JL came up with the study idea, performed the statistical analyses and drafted the manuscript. KH, GH, and KT contributed continuously to the manuscript throughout the writing process. All authors read and approved the final manuscript.

## Pre-publication history

The pre-publication history for this paper can be accessed here:

http://www.biomedcentral.com/1471-2458/13/545/prepub
